# Evidence-based practice among Italian osteopaths: a national cross-sectional survey

**DOI:** 10.1186/s12906-021-03430-y

**Published:** 2021-10-07

**Authors:** Francesco Cerritelli, Alessio Iacopini, Matteo Galli, Oliver P. Thomson, Tobias Sundberg, Matthew J. Leach, Jon Adams

**Affiliations:** 1 Clinical-based Human Research Department, Foundation COME Collaboration, Pescara, Italy; 2 University College of Osteopathy, London, UK; 3 Australian Research Centre in Complementary and Integrative Medicine (ARCCIM), Faculty of Health, University of Technology Sydney, Sydney, NSW Australia; 4 Musculoskeletal and Sports Injury Epidemiology Center (MUSIC), Department of Health Promotion Sciences, Sophiahemmet University, Stockholm, Sweden; 5 Unit of Intervention and Implementation Research on Worker Health, Institute of Environmental Medicine, Karolinska Institutet, Stockholm, Sweden; 6 National Centre for Naturopathic Medicine, Southern Cross University, Lismore, NSW Australia

**Keywords:** Evidence-based practice, Osteopathy, Attitude, Skill, Use, Cross-sectional studies

## Abstract

**Background:**

While evidence-based practice (EBP) is widely accepted across healthcare professions, research investigating its implementation in manual therapy professions such as osteopathy is limited. The primary aim of this study was to investigate Italian osteopaths’ attitudes, skills, and use of EBP. A secondary purpose was to understand the obstacles and enablers to EBP adoption in the Italian osteopathic context.

**Methods:**

A cross-sectional national survey was conducted (April to June 2020) among a sample of Italian osteopaths. Eligible participants were invited to complete the Italian-translated Evidence-Based practice Attitude and Utilization Survey (EBASE) anonymously online using various recruitment strategies, including email and social media campaigns. In addition to the three EBASE sub-scores (attitudes, skills and use), the demographic characteristics of the sample were considered.

**Results:**

A total of 473 osteopaths responded to the survey. The sample appeared to represent the Italian osteopathic profession. The majority of participants had a favorable attitude toward EBP. Eighty-eight percent of respondents agreed that EBP was necessary for osteopathy practice and that scientific literature and research findings were beneficial to their clinical scenario (95%). Perceived skill levels in EBP were rated as moderate, with the lowest scores for items relating to clinical research and systematic review conduct. Apart from reading/reviewing scientific literature and using online search engines to locate relevant research papers, participant engagement in all other EBP-related activities was generally low. Clinical practice was perceived to be based on a very small proportion of clinical research evidence. The primary obstacles to EBP implementation were a dearth of clinical evidence in osteopathy, and poor skills in applying research findings. The primary enablers of EBP adoption were access to full-text articles, internet connectivity at work, and access to online databases.

**Conclusions:**

Italian osteopaths were largely supportive of evidence-based practice but lacked basic skills in EBP and rarely engaged in EBP activities. The updating of osteopathic training curriculum and professional formal regulation in Italy could provide a suitable framework to improve EBP skills and use.

## Background

Evidence-based practice (EBP) was developed to improve the outcomes of therapies delivered within the healthcare system [1–3] posited as an optimal method for delivering best-practice care since the 1990s. By integrating the best available evidence with the practitioner’s clinical expertise and the patients’ values and preferences, EBP focuses its attention on the patient’s individuality within the context of their lives [3, 4].

While the intended aim of EBP is to improve the safety and efficacy of healthcare interventions, it does pose a challenge for professions where there is a paucity of evidence for their respective disciplines or when practitioners perceive that there is a conflict between EBP and their person-centred models of practice [5]. Decision-making pathways guided by defined algorithms can be interpreted as limiting the therapist’s autonomy, leading to applying rigid care protocols. Consideration also should be given to the potential limitations of EBP within the context of individual or complex clinical frameworks. This issue is particularly relevant to disciplines such as osteopathy, where the evaluation of an individual patient’s adaptive capacity potentially informs the selection of the structure-function model of approach and the consequent therapeutic focus [6].

Osteopathy is a manual therapy based on principles and concepts emphasizing the integration of structure and function and the harmonious functioning of neurological, musculoskeletal, circulatory and visceral structures within a psychosocial context [7, 8]. However, there is a growing understanding that osteopaths’ values, conceptions, and views regarding traditional osteopathic theory and principles vary [9, 10]. Most of these osteopathic principles were derived decades ago through clinical observations and interpretations of prominent individuals [11, 12]. Esteves et al. (2020) underline that many of these models are now outdated, suggesting that osteopathic practice needs to be better informed by the latest findings of osteopathic research [12]. That research evidence provides an opportunity to support, develop and potentially revise osteopathic principles and practice, so they serve the interests of patients by removing outdated models of practice or ineffective treatments [11, 13]. Fear that research evidence may contradict or fail to support traditional osteopathic principles has been found to be a barrier to the uptake of EBP in the international osteopathic community [11, 14].

The local/national professional context also has a strong influence on the clinical decision-making process of osteopaths, including to what extent that clinical decisions are made within an EBP framework. According to the survey conducted by Cerritelli et al. (2019), Italian professionals comprise approximately 4800 qualified osteopaths who have obtained a Diploma in Osteopathy (DO), which includes attending mainly part-time courses held by private institutions, following the achievement of previous academic degrees (sports science and physiotherapy) [15]. While the profession of osteopathy is recognised in Italy, with the publication of the professional profile by the competent ministerial bodies [16], it is not yet fully regulated.

While recent studies have shed light on the skills, attitudes and use of EBP amongst osteopaths in the UK [11], Sweden [17], Australia [18] and Spain [19], there have been no such studies conducted on osteopaths in Italy. As such, this study aimed to address this knowledge gap and investigate the attitudes, skills and use of evidence-based practice among Italian osteopaths.

## Methods

### Design

National, cross-sectional survey of Italian osteopaths.

### Participants

The sample size calculation was based on a target population composed of approximately 4800 osteopaths [15]. The number of participants was reached considering the method proposed by Vet et al. [20], whereby 6 participants were required for each questionnaire item. Taking into account possible drop-outs, the number was incremented by 10%, resulting in a required sample size of 475. The inclusion criteria were completion of the Diploma in Osteopathy (DO), according to the guidelines set out in the CEN Norm, for The European Standard on Osteopathic Healthcare Provision [21].

### Description of questionnaire and variables

The Evidence-based practice Attitude and utilization SurvEy (EBASE) is an 84-item tool that assesses attitudes, perceived abilities, and use of EBP in healthcare professionals. EBASE has undergone psychometric evaluation and has been shown to have good internal consistency, content validity, construct validity and test-retest reliability [11, 22]. The survey comprises seven parts: attitude (Part A), skills (Part B), education and training (Part C), use (Part D), barriers to EBP (Part E), EBP enablers (Part F), and participant demographics (Part G). Three of these parts can generate subscores, including Attitudes subscore (i.e. the sum of 6 items from Part A, with scores ranging from 8 [predominantly strongly disagree] to 40 [predominantly strongly agree]); Skill subscore (i.e. the sum of all 13 items from Part B, with scores ranging from 13 [primarily low-level skill] to 65 [primarily high-level skill]); and Use subscore (i.e. the sum of 6 items from Part D, with scores ranging from 0 (mainly infrequent use) to 24 (mainly frequent use).

Because the EBASE questionnaire was originally designed for Australian CAM practitioners, some items required modification to ensure the questionnaire was replicable for the Italian osteopathy population, as has been done in previous EBASE studies [11, 19]. For instance, demographic items in part G of the questionnaire were altered to be geographically relevant. The term “CAM” was also replaced with “osteopathy” throughout the questionnaire. These changes did not alter the intent of the items.

The EBASE questionnaire was translated into Italian following established guidelines [23, 24]. Two native Italian people speaking fluent English independently translated EBASE. One of the two was a professional English-Italian translator with 20 years of experience but without medical experience; the other was an Italian medical professional. The two translators agreed on a concordant version of the translated questionnaire, which was then independently back-translated by two English native speakers fluent in Italian. An expert committee comprising translators, a linguistic expert, two osteopaths, and one epidemiologist discussed the final translation and analysed it from a semantic, idiomatic, experiential, and conceptual point of view. At this point, the Italian version of EBASE (EBASE-I) was tested on an 18-year old reader that fully understood the text.

The EBASE-I was then pilot-tested on a convenience sample of 10 Italian osteopaths (DOs) to determine whether survey items were clear, complete and appropriate. DOs were recruited primarily via members of the Foundation COME Collaboration, with each participant reporting a different level of education and clinical experience. Three authors (FC, AI, MG) reviewed the participant feedback, which resulted in amendments to several unclear words and issues related to the comprehensibility of some items. The estimated completion time of the questionnaire was 10–15 min.

### Data collection

Potential participants were invited to participate in the survey through emails sent by the Foundation COME Collaboration. A reminder email was sent to osteopaths 15 days later. The survey was also promoted via social media and Italian websites reporting osteopathic news. All emails and online posts contained an invitation letter, a link to the questionnaire (which was hosted by Google Forms), a participant information sheet and a consent form, which participants had to accept and digitally sign before completing the survey. Participants were only able to complete the survey once. Data collection was undertaken between April 2020 and June 2020.

### Statistical analysis

Survey responses were imported into R version 3.5.1 (R core Team 2017) and Rstudio Version 1.1.463 (RStudio, Inc. 2009–2018) for data analysis. There were no missing data as all items were made compulsory. The level of significance was set at < 0.05. Different measures of central tendency were used to describe the data, where appropriate, including mean, median, mode, point estimates, range, standard deviation, and 95% confidence intervals. The internal consistency of EBASE-I was measured using Cronbach’s alpha.

A linear regression model explored correlations between demographic variables (e.g. hours dedicated to osteopathic clinical practice weekly, hours dedicated to research weekly) and EBASE-I scores. Selection of the model was determined using regsubsets() function of R package “leaps”, which returns the best models and their adjusted R2. The final model was selected by considering the highest adjusted R2. Receiver Operating Characteristic (ROC) analysis and Area Under the Curve (AUC) calculations were performed to confirm the goodness of fit for the model.

### Ethical approval and informed consent

Ethical approval was obtained from the Centre for Osteopathic Medicine Collaboration Institutional Review Board via an expedited review (10/2019). Study was carried out in accordance with ethical guidelines of Centre for Osteopathic Medicine institution. Informed consent was obtained from all the participants. Consent forms and participant data were stored on a dedicated, encrypted and protected server, according to the European directive 2018/1725/CE of the European Parliament. Individual surveys were also vetted to ensure participants could not be identified. Only those who performed the analysis had access to the data.

## Results

A total of 500 osteopaths were contacted, of which 475 (95%) responded. Excluding 2 responses that did not fulfil the inclusion criteria, the final sample size was 473 participants, which exceeded the required sample size.

### Demographic characteristics of the sample

Table 1 reported data on the study sample. The majority of participants were male (58%), aged over 30 years (*n* = 340, 72%), with the most prevalent age category being 30–49 years (*n* = 260, 55%). Most participants held a bachelor degree qualification or higher (76%), and most had practiced osteopathy for less than 10 years (73%), mainly in private practice (65%) and in the north-west of Italy (43%).

**Table 1:** Demographic characteristics of the sample (*n* = 473)

	*n (%)*
Gender (M)	274 (58)
Age	
20–29	133 (28)
30–49	260 (55)
50–60	80 (17)
Degree	
Diploma	133 (24)
Bachelor or higher	360 (76)
Working experience (less 10 years)	346 (73)
Context	
Private practice	307 (65)
Public practice	156 (33)
Formation (e.g. university, school)	10 (2)
Region (North-west)	202 (43)

### Attitudes towards EBP

Participants’ attitudes toward EBP were positive, with a median attitude subscale score of 31 (IQR 29, 34; range 8–40). Most of the sample agreed that EBP is necessary for practising osteopathy (88%), assists clinical decision making (78%), and is useful in day-to-day practice (90%) (Table 2). The majority of participants were also interested in learning or improving the skills necessary to incorporate EBP into their osteopathic practice (95%) (Table 2). Attitude items in EBASE-I demonstrated a good level of internal consistency (raw Cronbach’s alpha = 0.83; standardised Cronbach’s alpha = 0.84) (Table 3).

**Table 2:** Participant attitudes toward evidence-based practice (*n* = 473)

	*1* Strongly Disagree *n(%)*	*2* Disagree *n(%)*	*3* Neutral *n(%)*	*4* Agree *n(%)*	*5* Strongly Agree *n(%)*
Evidence-based practice is necessary in the practice of osteopathy	2 (0.4)	16 (3.4)	38 (8)	259(54.8)	158 (33.4)
Professional literature (ie: journals & textbooks) and research findings are useful in my day-to-day practice	2 (0.4)	10 (2.1)	37 (7.8)	262 (55.4)	162(34.3)
I am interested in learning or improving the skills necessary to incorporate evidence-based practice into my osteopathic practice	2 (0.4)	5 (1.1)	18 (3.9)	242 (51.1)	206 (43.5)
Evidence-based practice improves the quality of my patient’s care	4 (0.8)	18 (3.9)	90 (19)	238 (50.3)	123 (26)
Evidence-based practice assists me in making decisions about patient care	3 (0.6)	19 (4)	81 (17.2)	245 (51.8)	125 (26.4)
Evidence-based practice takes into account my clinical experience when making clinical decisions	4 (0.8)	22 (4.7)	78 (16.5)	269 (56.9)	100 (21.1)
Evidence-based practice takes into account a patient’s preference for treatment	10 (2.1)	111 (23.5)	151 (31.9)	146 (30.9)	55 (11.6)
The adoption of evidence-based practice places an unreasonable demand on my practice	33 (7)	237 (50.1)	144 (30.4)	49 (10.4)	10 (2.1)
There is a lack of evidence from clinical trials to support most of the treatments I use in my practice	14 (3)	133 (28.1)	107 (22.6)	179 (37.8)	40 (8.5)
Prioritizing evidence-based practice within osteopathic practice is fundamental to the advancement of the profession	8 (1.7)	34 (7.2)	60 (12.6)	197 (41.7)	174 (36.8)

**Table 3:** Internal consistency of EBASE-I

	Cronbach’s alpha (raw)	Cronbach’s alpha (standardised)
Attitudes	0.83	0.84
Skill in EBM	0.92	0.92
Use of EBM	0.84	0.84

Multivariate linear analysis demonstrated statistically significant associations between EBP attitudes and gender, dedicating at least 1 h per week in research, being part of a professional association, having published, and being familiar with the English language (Table 4).

**Table 4:** Attitudes score multivariate linear analysis

Characteristics	β	95% C.I.	*P*-value
Age 30–49	−0.86	−1.80 to 0.08	*p* = 0.07
Age 50–59	−0.93	−2.51 to 0.66	*p* = 0.25
Gender (M)	1.02	0.25 to 1.80	***p*** **< 0.01**
Working Experience (0–10 years)	−0.1	−1.33 to 1.13	*p* = 0.87
Working Experience (11 or more years)	−0.94	−2.45 to 0.57	*p* = 0.22
Having dedicate at least an hour in research in week (yes)	1.13	0.33 to 1.93	***p*** **< 0.01**
Being part of an Association (yes)	1.49	0.64 to 2.34	***p*** **< 0.001**
Having at least one publications (yes)	1.55	0.47 to 2.62	***p*** **< 0.01**
English language (yes)	1.33	0.52 to 2.15	***p*** **< 0.01**

The level of significance was set at < 0.05

### Skills in EBP

Responders reported moderate levels of perceived skill in EBP, with a median skills subscore of 38 (IQR 33,45; range 13–64). Participants reported high levels of skill for identifying knowledge gaps in practice, considering answerable clinical questions, and sharing evidence with colleagues (Table 5). The lowest skill levels were reported for the conduct of clinical research and systematic reviews (Table 5). Internal consistency across all EBASE skills items was good (raw Cronbach’s alpha = 0.92; standardised Cronbach’s alpha = 0.92).

**Table 5:** Participant self-reported skills in evidence-based practice (*n* = 473)

	*1* Low *n(%)*	*2* Low-moderate *n(%)*	*3* Moderate *n(%)*	*4* Moderate-High *n(%)*	*5* High *n(%)*
Identifying knowledge gaps in practice	16 (3.4)	51 (10.8)	229 (48.4)	156 (33)	21(4.4)
Identifying answerable clinical questions	4 (0.8)	40 (8.4)	180(38.1)	227 (48.0)	22 (4.6)
Locating professional literature (i.e. journal articles)	24 (5.1)	78 (16.5)	157 (33.2)	142 (30)	72 (15.2)
Online database searching (e.g. PubMed)	28 (5.9)	75 (15.9)	144 (30.4)	148 (31.3)	78 (16.5)
Retrieving evidence	32 (6.8)	113 (23.9)	163 (34.5)	124 (26.2)	41 (8.7)
Critical appraisal of evidence	70 (14.8)	135 (28.5)	146 (30.9)	95 (20.1)	27 (5.7)
Synthesis of research evidence	57 (12.1)	94 (19.9)	183 (38.6)	112 (23.7)	27 (5.7)
Applying research evidence to patient cases	18 (3.8)	72 (15.2)	208 (44)	151 (31.9)	24 (5.1)
Sharing evidence with colleagues	47 (9.9)	73 (15.4)	138 (29.2)	163 (34.5)	52 (11)
Conducting clinical research (e.g. clinical trials)	173 (36.6)	117 (24.7)	115 (24.3)	48 (10.2)	20 (4.2)
Using findings from clinical research	50 (10.6)	130 (27.5)	193 (40.8)	78 (16.5)	22 (4.6)
Conducting systematic reviews	168 (35.5)	128 (27.1)	106 (22.4)	61 (12.9)	10 (2.1)
Using findings from systematic reviews	60 (12.7)	114 (24.1)	165 (34.9)	111 (23.5)	23 (4.8)

The best model for multivariate analysis found statistically significant associations between skills subscale score and participant age, highest level of education, having knowledge of the English language, being involved in research at least 1 h per week, having published, and being a member of a professional association (Table 6).

**Table 6:** Skill score multivariate linear analysis

Characteristics	β	95% C.I.	*P*-value
Age 30–39	−2.31	−4.26 to −0.36	***p*** **< 0.05**
Age 50–59	−3.23	−5.76 to −0.70	***p*** **< 0.05**
University Degree	2.92	0.98 to 4.85	***p*** **< 0.01**
Teacher in PG (yes)	2.07	−0.002 to 4.14	*p* = 0.05
Having dedicate at least an hour in Research in week (yes)	6.56	5.04 to 8.08	***p*** **< 0.001**
Being part of an Association (yes)	1.79	0.18 to 3.41	***p*** **< 0.05**
Having at least one publication (yes)	6.58	4.47 to 8.69	***p*** **< 0.001**
English language (yes)	5.01	3.46 to 6.56	***p*** **< 0.001**

The level of significance was set at < 0.05

### Use of EBP

Participants responding to the survey engaged in EBP activities at a moderate level in the 30 days preceding study enrollment, with findings revealing a median use subscore of 13 (IQR 13,17; range 6–30). More than 64% of participants read/reviewed/used professional literature or research findings relevant to their practice at least once in the preceding month (Table 7). One-half (52%) of participants consulted a colleague to assist their clinical decision-making 1-to-5 times in the previous month, and 42% used an online search engine with the same frequency (Table 7). In terms of the extent to which clinical trial evidence informed participant practice, one-third of participants (32%) rated the extent as poor, 31% as fair, 26% as moderate, and 8% as substantial. The internal consistency of EBP use items was found to be good (raw Cronbach’s alpha = 0.84; standardised Cronbach’s alpha = 0.84).

**Table 7:** Participant use of evidence-based practice (i.e. number of times each activity was performed over the last month) (*n* = 473)

	*1* 0 times *n(%)*	*2* 1–5 times *n(%)*	*3* 6–10 times *n(%)*	*4* 11–15 times *n(%)*	*5* 16+ times *n(%)*
I have read/reviewed professional literature (i.e. professional journals & textbooks) related to my practice	57 (12.1)	214 (45.2)	105 (22.2)	37 (7.8)	60 (12.7)
I have read/reviewed clinical research findings related to my practice	97 (20.5)	226 (47.8)	81 (17.1)	28 (5.9)	41 (8.7)
I have used professional literature or research findings to assist my clinical decision-making	113 (23.9)	245 (51.8)	74 (15.6)	14 (3)	27 (5.7)
I have used professional literature or research findings to change my clinical practice	170 (35.9)	229 (48.4)	52 (11)	9 (1.9)	13 (2.8)
I have used an online database (e.g. PubMed, MEDLINE) to search for practice related literature or research	111 (23.5)	184 (38.9)	75 (15.9)	41 (8.6)	62 (13.1)
I have used an online search engine (e.g. Google) to search for practice related literature or research	104 (22)	198 (41.9)	78 (16.5)	45 (9.5)	48 (10.1)
I have consulted a colleague or industry expert to assist my clinical decision-making	151 (31.9)	247 (52.2)	56 (11.9)	12 (2.5)	7 (1.5)
I have referred to magazines, layperson/self-help books, or non-government/non-education institution websites to assist my clinical decision-making	242 (51.2)	180 (38.1)	33 (7.0)	5 (1.0)	13 (2.7)

Multivariate analysis showed that use of EBP was statistically significantly associated with gender, being involved in research for at least 1 h per week, having published at least 1 scientific paper in a peer-reviewed journal, teaching in post-graduate courses and being familiar with the English language (Table 8).

**Table 8:** Use score multivariate linear analysis

Characteristics	β	95% C.I.	*P*-value
Gender (M)	1.57	0.76 to 2.38	*p* < 0.001
University Degree	0.60	−0.34 to 1.55	*p* = 0.21
Having dedicate at least an hour in Research in week (yes)	3.70	2.878 to 4.51	*p* < 0.001
Context (private practice)	0.38	−2.35 to 3.12	*p* = 0.78
Context (public practice)	1.04	− 1.74 to 3.82	*p* = 0.46
Area (metropolitan)	0.66	−0.33 to 1.65	*p* = 0.19
Having at least one publication (yes)	1.99	0.83 to 3.14	*p* < 0.001
Teacher in PG (yes)	1.27	0.17 to 2.37	*p* < 0.05
English language (yes)	1.69	0.85 to 2.53	*p* < 0.001

The level of significance was set at < 0.05

### Training in EBP

Most participants reported some level of training in EBP (87%), which was largely developed during their undergraduate programmes (22%). Lower rates of training were reported in areas such as critical thinking/analysis (68%) and clinical research (77%). One-half (50%) of participants learned EBP skills from a post-graduate course or from a short-term educational program. More than one-half (53%) of participants reported having no training in conducting systematic reviews and over one-third (38%) reported no training in clinical trials.

### Barriers and enablers of EBP uptake

The lack of research evidence in osteopathy (71%), and lack of time (60%) were reported as moderate to major barriers to EBP uptake by most participants. By contrast, the majority of participants (86.5%) did not report patient preferences for treatment as a barrier to EBP uptake. Internet in the workplace (60.8%), ability to download full-text articles (61.7%), and access to free online databases (68.2%) and online EBP education materials (61.5%) were identified by most participants as being ‘very useful’ enablers of EBP. Access to critical reviews (87.1%), databases requiring licence fees (90.8%), and critically appraised topics relating to osteopathy (94.5%) were reported as “moderately to very useful” enablers of EBP by the majority of participants (Table 9).

**Table 9:** Enablers to EBP (*n* = 473)

	*1* Not useful *n(%)*	*2* Slightly useful *n(%)*	*3* Moderately useful *n(%)*	*4* Very useful *n(%)*
Access to the Internet in your workplace	21 (4.4)	44 (9.3)	120 (25.4)	288 (60.9)
Access to free online databases in the workplace, such as Cochrane and PubMed	16 (3.4)	30 (6.3)	103 (21.8)	324 (68.5)
Free access to online databases that usually require license fees, such as MEDLINE and CINAHL	19 (4)	24 (5.1)	112 (23.7)	318 (67.2)
Ability to download full-text / full-length journal articles	12 (2.5)	29 (6.2)	140 (29.6)	292 (61.7)
Access to online education materials related to evidence based practice	6 (1.3)	21 (4.4)	154 (32.5)	292 (61.7)
Access to tools used to assist the critical appraisal / evaluation of research evidence	6 (1.3)	51 (10.8)	187 (39.5)	229 (48.4)
Access to critically appraised topics relevant to osteopathy (these are critical appraisals of single research papers)	3 (0.6)	32 (6.8)	188 (39.7)	250 (52.9)
Access to critical reviews of research evidence relevant to osteopathy (these are critical reviews of multiple research papers addressing a single topic)	5 (1.1)	19 (4)	163 (34.4)	286 (60.5)
Access to research rating tools that facilitate critical appraisal of single research papers	6 (1.3)	53 (11.2)	179 (37.8)	235 (49.7)
Access to online tools that assist you to conduct your own critical appraisals of multiple research papers related to a single topic	10 (2.1)	55 (11.6)	164 (34.7)	244 (51.6)

## Discussion

The present study aimed to investigate the attitudes, skills and use of evidence-based practice among Italian osteopaths. Our results suggest that Italian osteopaths have favorable views of EBP, considering it necessary for their clinical practice and the osteopathic profession’s development. Although the required sample size was achieved, our sample might only represent 10 % of the entire osteopathic population in Italy [15, 25]. For example, our sample of participants had a high proportion of males and those aged 30–39 years, similar to data from a recent survey of Spanish osteopaths [19]. Previous studies using EBASE have also reported gender imbalances [11, 18]. Notwithstanding, our findings were comparable to those reported in studies of Spanish and UK osteopaths [11, 19].

### Attitudes towards EBP

Most participants expressed satisfaction with using EBP to improve clinical outcomes, which aligned with attitudes reported amongst Spanish osteopaths [19]. However, while participants reported positive attitudes toward using scientific literature to assist with decision making, the utilization of such evidence in clinical practice was low. This pattern is consistent with that observed in studies of other health disciplines, including chiropractic, osteopathy, naturopathy, traditional Chinese medicine, physical therapy/physiotherapy and nursing [11, 26–29]. In our study, this mismatch between attitude and practice behaviour may relate to some extent to the perceived lack of evidence in the osteopathic field. Indeed, almost half of our participants reported this as a concern, which appears to assume greater significance than that expressed by participants from other CAM disciplines [27, 28]. A recent review found that trial-based evidence in osteopathy is of poor quality (e.g. inadequate sample size, study length, and controls), and the quality of reporting has not improved over time [30]. Accordingly, an evident change in strategy and continued effort has been recommended to promote rigorous methodology in the design of future osteopathic research [30].

A noteworthy finding was the positive association between confidence with the English language and attitude towards EBP. An analysis of this data might suggest two possibilities: first, EBP is primarily a concept used in English-language cultures, as postulated by Hunt & Ernst [31]. Second, most scientific publications are reported in English. Thus, having poor English-language proficiency may prevent Italian osteopathic practitioners from fully implementing EBP into routine clinical practice. It is likely that, even though there are few precedents, there is little or no institutional attention paid to the incorporation of EBP paradigm into clinical decision-making processes, promoting educational paths to support the linguistic interpretation of data, the use of evidence and the design of studies with rigorous methodology both within the private and public under- and post- graduate training sectors [32].

### Skills and use

The majority of participants were comfortable with identifying clinical questions that could be answered through research. This might be because participants have a basic understanding of the most common clinical conditions [25, 33]. The application of the steps of the EBP process appears contentious. Indeed, more confidence was found in the evidence application to the clinical context - the second to last step of the EBP process - rather than with the data retrieval, critical evaluation, and synthesis. Moreover, the data suggested that the awareness of practical skills in the conduct and use of data observed during clinical trials and systematic reviews is scarce. It is possible that the scarcity of the osteopathic sample culture analyzed with regard to the EBP paradigm is related to the observed phenomena. We can assume that a lack of EBP context awareness is one of the reasons for the clinical inefficiency in incorporating evidence.

Higher education degrees, involvement in research activities, and publication in scientific journals are the foundation for evidence-based practices within clinical environments. However, the absolute numbers suggest that engagement in research and scientific publication remains relatively limited, with most research and publication being confined to a small niche.

Interestingly, the older you get, the less useful skills seem to be, confirming data on the attitude domain.

Regarding the use of EBP, data clearly showed that over one-third of responses barely use the EBP. Additionally, 24% of participants did not utilize the internet to find scientific data, and the process of care was not informed by reading scientific literature, although 73% of the sample declared to be in the workforce for less than 10 years. The rational treatment process and selection for manipulative approaches appears to be conditioned by cultural elements derived from the osteopathic tradition. This appears to follow the findings in other countries, such as the UK [11], Australia [18], Sweden [17], and Spain [19]. Our best explanation is that the approach and curriculum that the Italian schools of osteopathy are using to train new osteopaths is based on a method of teaching in which the teacher acts as an expert and a mentor, helping the students learn through a hands-on approach.

Furthermore, the use of EBP exhibited a gender association, as males were more likely to use it. Despite the lack of evidence in CAM literature regarding this association, in other healthcare professions, gender differences are described that support our findings [34, 35].

Conversely, research activities and research publications appear associated with a greater likelihood of evidence-based practice being integrated into the clinical context, showing that the more research-like activities one takes on, the more positive is the effect on EBP attitude [32].

Interestingly, research analysis shows that teaching in post-graduate courses appears to be linked to using EBP. This describes that lecturers are more likely to incorporate EBP in their clinical practice, supporting EBP competency in students [32, 36].

### Enablers, barriers and training in EBP

The sample expressed a need for greater access to research literature as an important enabler of EBP uptake; this included access to the internet, online bibliographic databases, free full-text articles, and educational material on EBP. By contrast, participants reported lack of time and lack of clinical evidence in the osteopathic field as the main obstacles to applying EBP.

Interestingly, none of the major barriers to EBP uptake was linked to training, even though participants did indicate elsewhere that there was a need for skill development in some areas of EBP (e.g. acquiring and appraising the evidence). We would argue that the design of educational paths clearly conditions the development of expertise on the application of scientific evidence in clinical contexts. Academic institutions should change the educational pathway by investing in evidence-based and informed practices. Indeed, most of the participants in the study have recently graduated (73% declared less than 10 years of working experience), but their EBP skills appear limited. Since the publication of the CEN Osteopathic Healthcare Provision Norm in 2015 [21], efforts have been made in several national contexts to reform the subjects taught, focusing on raising their level of scientific accuracy. It will take time for this evolutionary process to take place due to the required shifts in training plans, strategies, and teaching styles. Indeed, we believe that the first step in implementing a strategic change must occur within training institutions. Boards of directors, together with their teaching staff and the new curricula structure, should hopefully provide educational programs that incorporate evidence-based training in both theoretical and clinical contexts, driving a cultural shift in EBP training in osteopathic formation and impact of teaching on participants’ EBP competency, consisting of critical appraisal skills, knowledge and/or behaviour [36].

### Limitations

While EBASE is a validated instrument, the psychometric properties of the Italian version of the questionnaire have not been formally assessed, which could have impacted the study’s findings. However, it should be noted that translation of the questionnaire was conducted following WHO procedures [24], and piloted, and Cronbach’s alpha demonstrated a high level of internal consistency. Other inherent limitations of the study design include recall bias and selection bias.

## Conclusions

This study elucidates the attitudes, skills, and practices of Italian osteopaths about EBP. The attitudes of participants toward EBP were positive. At least four out of five participating osteopaths agreed that EBP was needed in their routine clinical practice. Nonetheless, the perceived skill level and use of EBP was found to be moderate. However, there was a common interest in acquiring and developing the skills required to implement EBP in clinical practice. Based on our findings, we argue that incorporating osteopathic educational programs into university environments alongside a pedagogical shift that promotes critical thinking and reflective practice will benefit Italian osteopaths in embracing EBP; now that, in Italy, osteopathy has been recognised as a healthcare profession and included in academic training.

## Data Availability

The datasets used and/or analysed during the current study are available from the corresponding author on reasonable request.

## Abbreviations

EBP: Evidence-based practice
CAM: Complementary and alternative medicine
EBASE: Evidence-Based practice Attitude and utilization SurvEy
WHO: World Health Organization
IQR: Interquartile range
